# A general one-pot strategy for the synthesis of Au@multi-oxide yolk@shell nanospheres with enhanced catalytic performance†
†Electronic supplementary information (ESI) available. See DOI: 10.1039/c8sc01520a


**DOI:** 10.1039/c8sc01520a

**Published:** 2018-08-06

**Authors:** Jian Li, Shuyan Song, Yan Long, Shuang Yao, Xin Ge, Lanlan Wu, Yibo Zhang, Xiao Wang, Xiangguang Yang, Hongjie Zhang

**Affiliations:** a State Key Laboratory of Rare Earth Resource Utilization , Changchun Institute of Applied Chemistry , Chinese Academy of Sciences , Changchun 130022 , P. R. China . Email: songsy@ciac.ac.cn ; Email: yibozhang@ciac.ac.cn ; Email: hongjie@ciac.ac.cn; b School of Applied Chemistry and Engineering , University of Science and Technology of China , Hefei 230026 , Anhui , P. R. China

## Abstract

By integrating redox self-assembly and redox etching processes, we report a general one-pot strategy for the synthesis of Au@multi-M_*x*_O_*y*_ (M = Co, Ce, Fe, and Sn) yolk@shell nanospheres. Without any additional protecting molecule or reductant, the whole reaction is a clean redox process that happens among the inorganic metal salts in an alkaline aqueous solution. By using this method, Au@Co_3_O_4_/CeO_2_ (Au@Co–Ce), Au@Co_3_O_4_/Fe_2_O_3_ (Au@Co–Fe), and Au@CeO_2_/SnO_2_ (Au@Ce–Sn) yolk@shell nanospheres with binary oxides as shells, Au@Co_3_O_4_/CeO_2_/Fe_2_O_3_ (Au@Co–Ce–Fe) yolk@shell nanospheres with ternary oxides as shells and Au@Co_3_O_4_/CeO_2_/Fe_2_O_3_/SnO_2_ (Au@Co–Ce–Fe–Sn) yolk@shell nanospheres with quaternary oxides as shells can be obtained. Subsequently, the catalytic CO oxidation was selected as the catalytic model, and the Au@Co–Ce system was chosen as the catalyst. It was found that the catalytic activity of Au@Co–Ce yolk@shell nanospheres can be optimized by altering the relative proportion of Co and Ce oxides.

## Abstract 2

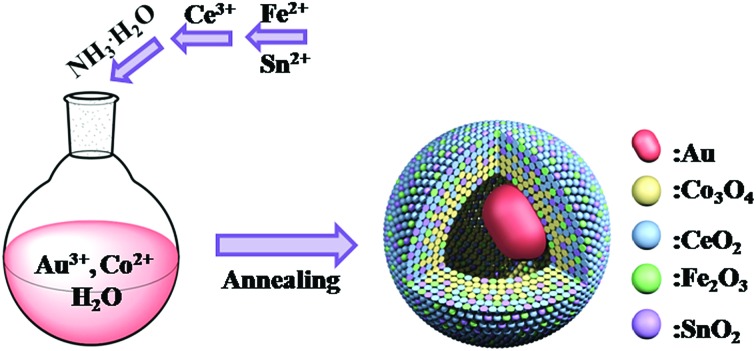
By integrating redox self-assembly and redox etching processes, we report a general one-pot strategy for the synthesis of Au@multi-M_*x*_O_*y*_ (M = Co, Ce, Fe, and Sn) yolk@shell nanospheres.

## Introduction

Compared with single component nanomaterials, hybrid nanomaterials with complex compositions may exhibit enhanced physical and chemical properties based on the synergistic effect principle.^1^,^2^ Furthermore, altering the relative proportion of different compositions in hybrid nanomaterials can provide a new approach for optimizing their properties.^3^,^4^ As one of the ideal patterns for hybrid nanomaterials, noble metal@oxide yolk@shell nanospheres (YSNs) have intriguing properties, such as low density, high surface area, and interstitial hollow spaces, leading to their potential applications in the fields of photothermal therapy,^5^ gas sensing,^6^ and drug release,^7^ especially catalysis.^8^ For their preparation, numerous approaches have been developed based on template-assisted processes, the Kirkendall effect, and Ostwald ripening.^9^–^11^ However, most of these methods are only suitable for YSNs with single oxide composition. For YSNs with shells containing multiple oxides (MOYSNs), these methods usually involve complicated fabrication processes, which seriously impede their practical applications.^11^ Therefore, challenges still exist in the development of facile and clean methods to fabricate MOYSNs with enhanced performance.^11^

Recently, based on the principle of the auto-catalytic redox reaction followed by a spontaneous self-assembly process, a green strategy has been developed by our group and others to fabricate CeO_2_-encapsulated noble metal core@shell nanostructures.^12^–^15^ Both the core and shell are clean self-assembled together without a complicated experimental procedure, which are beneficial for the further optimization of the catalytic performance. Furthermore, binary oxide nanostructures have also been exploited by redox etching reactions between metal oxides (involving Ce, Co, Fe, Sn, and Mn elements).^16^–^18^ Herein, by integrating the redox self-assembly process and redox etching process, we report a general one-pot strategy for the synthesis of Au@multi-M_*x*_O_*y*_ (M = Co, Ce, Fe, and Sn) YSNs. The composition of the shell can be continuously adjusted from two components (Co_3_O_4_/CeO_2_, Co_3_O_4_/Fe_2_O_3_ or CeO_2_/SnO_2_) to four components (Co_3_O_4_/CeO_2_/Fe_2_O_3_/SnO_2_) by mixing HAuCl_4_ with the corresponding metal salts in the presence of NH_3_·H_2_O. The relative contents of the different metal oxides in nanospheres could be tuned by precisely controlling the reaction conditions. We note that the entire preparation processes are very simple and do not use any organics, providing a clean surface for further catalytic exploitation of the well-defined MOYSNs.

## Results and discussion

### The formation mechanism and characterization of the nanospheres

**Scheme 1:**
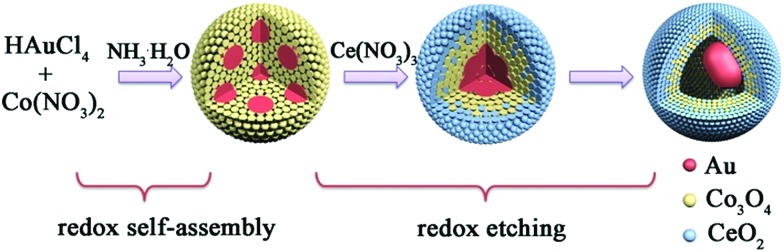
Schematic view of the formation process of Au@Co–Ce YSNs.

Taking the Au@Co–Ce sample for example, the formation process of MOYSNs is illustrated in Scheme 1. Essentially, the preparation of MOYSNs consists of the redox self-assembly process and then the *in situ* redox etching process. First, the Au^3+^ can oxidize Co^2+^ under alkaline conditions to trigger the redox assembly process, resulting in the formation of Au@Co_3_O_4_ nanospheres.^14^ Then the Co^3+^ in Co_3_O_4_ (Co^3+^/Co^2+^ = 1.92 V) shows strong oxidizability and can directly react with reducing ions Ce^3+^ (Ce^4+^/Ce^3+^ = 1.44 V), to yield binary oxide structures as shells.^14^,^16^–^19^ The ratio of Co and Ce could be tuned by precisely controlling the redox etching process. The mechanism of the formation of the YSN nanospheres has been discussed in the ESI section† according to their time-evolution TEM images and STEM-EDX elemental maps (Fig. S1†). Similarly, Ce^3+^ can be replaced with Fe^2+^ (Fe^2+^/Fe^3+^ = 0.77 V), Ce^3+^/Fe^2+^ and Ce^3+^/Fe^2+^/Sn^2+^ (Sn^4+^/Sn^2+^ = 0.15 V) to produce Au@Co–Fe, Au@Co–Ce–Fe, and Au@Co–Ce–Fe–Sn YSNs, respectively.^19^ Furthermore, based on the great difference of reduction potentials between Ce^4+^/Ce^3+^ and Sn^4+^/Sn^2+^, the Au@CeO_2_ nanospheres can also be etched by Sn^2+^ to yield the Au@Ce–Sn sample.

**Fig. 1:**
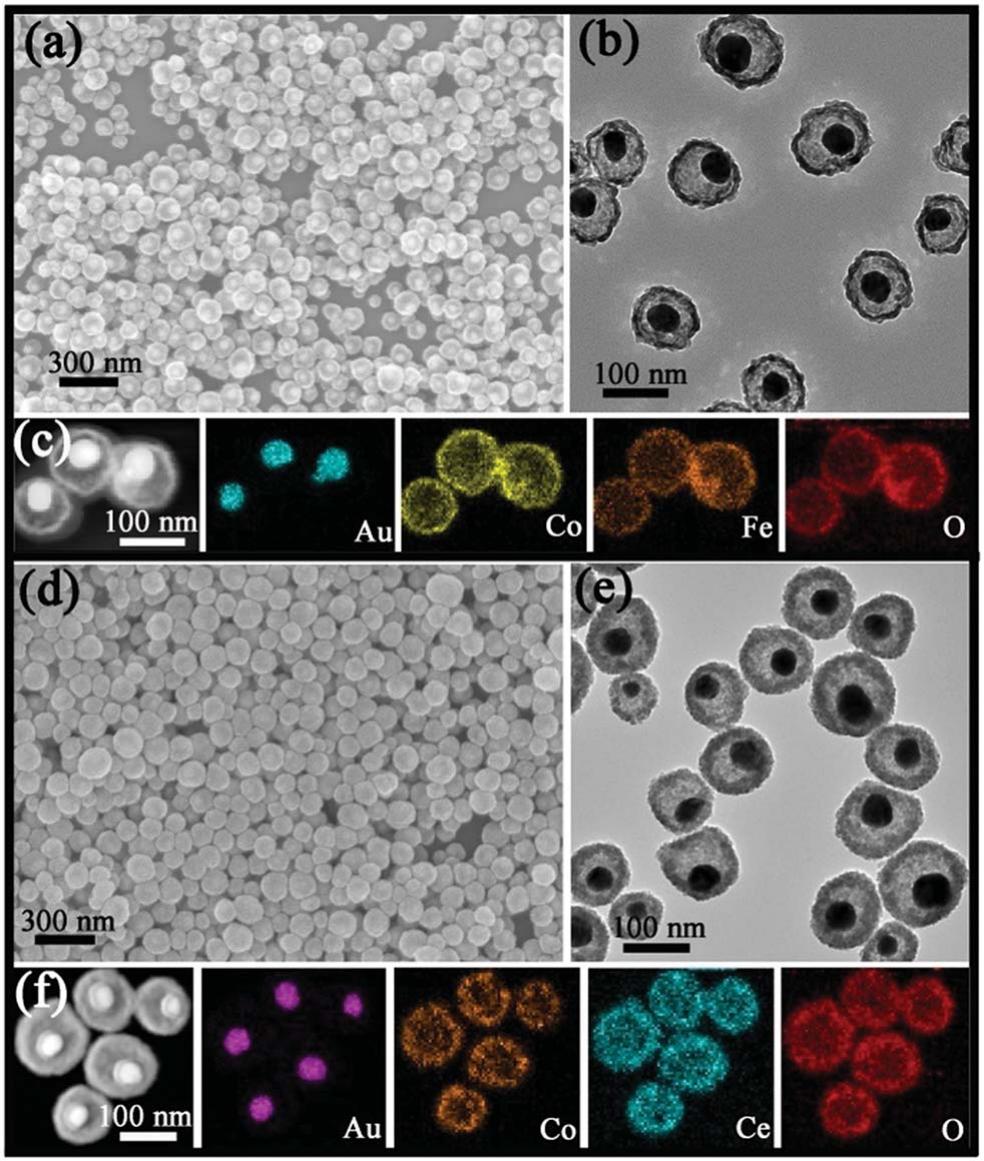
(a) SEM image, (b) TEM image and (c) STEM-EDX elemental maps of the Au@Co–Fe MOYSNs; (d) SEM image, (e) TEM image and (f) STEM-EDX elemental maps of the Au@Co–Ce MOYSNs.

In a typical experimental process, the Au@Co_3_O_4_ core@shell nanospheres were prepared by mixing HAuCl_4_, Co(NO_3_)_2_ and NH_3_·H_2_O for a certain time. And the detailed characterizations can be found in Fig. S2.† Furthermore, by directly adding FeCl_2_ or Ce(NO_3_)_3_ into the original solution of Au@Co_3_O_4_, Au@Co–Fe or Au@Co–Ce MOYSNs could be synthesized after further annealing for fine crystallization. The scanning electron microscopy (SEM) images in Fig. 1a and d reveal the uniform and monodisperse nanospheres of both samples with an average diameter of 105 nm. Furthermore, the transmission electron microscopy (TEM) images in Fig. 1b and e display the obvious yolk–shell features of both samples. It can be seen that Au nanoparticles with similar size (around 36 nm) in both samples are entirely encapsulated into the hollow shell. However, the shell in Fig. 1b is thinner than that in Fig. 1e. Because of the strong reducibility of Fe^2+^ and the fact that extra addition of the HCl solution into the Au@Co–Fe system can immensely accelerate the etching of Co_3_O_4_, nanospheres with a thinner shell are obtained. The energy-dispersive X-ray spectroscopy (EDX) elemental mappings (Fig. 1c and f) confirm the coexistence of two metal elements in the shells of both samples. Interestingly, Fe and Co are uniformly distributed in the whole shell in Au@Co–Fe MOYSNs (Fig. 1c). However, in Au@Co–Ce MOYSNs, Ce is present in the entire shell and Co is relatively distributed in the inner shell only. This might be caused by the direct deposition of a part of the CeO_2_ nanoparticles on the surface of the nanospheres in a relatively alkaline aqueous solution. However, the phenomenon cannot be observed in the Au@Co–Fe system due to the inhibition of the hydrolysis of Fe ions in a relatively acidic aqueous solution. Both of the samples were also examined by inductively coupled plasma (ICP) analysis. The average contents of Fe and Co in the Au@Co–Fe sample are 17.4 and 22.8 wt%, respectively. And the average contents of Ce and Co in the Au@Co–Ce sample are 47.4 and 19.8 wt%, respectively. The X-ray powder diffraction (XRD) pattern of the Au@Co–Ce sample is shown in Fig. S3.† All peaks can be perfectly indexed to metallic Au (JCPDS no. 04-0784), CeO_2_ (JCPDS no. 34-0394), and Co_3_O_4_ (JCPDS no. 42-1467). However, the XRD pattern (Fig. S4d†) of the Au@Co–Fe sample only shows the presence of metallic Au and Co–Fe binary oxides. Therefore, the Au@Co–Fe sample was further analyzed by X-ray photoelectron spectroscopy (XPS). The high-resolution XPS spectrum (Fig. S4b†) of Co shows two peaks at 781.2 and 796.7 eV, which are the characteristic peaks of Co 2p_3/2_, and Co 2p_1/2_ for Co_3_O_4_, respectively.^20^ In Fig. S4c,† the characteristic peaks of Fe 2p_3/2_ and Fe 2p_1/2_ for Fe_2_O_3_ are observed at the binding energies of 711.0 and 724.6 eV, respectively.^21^,^22^

**Fig. 2:**
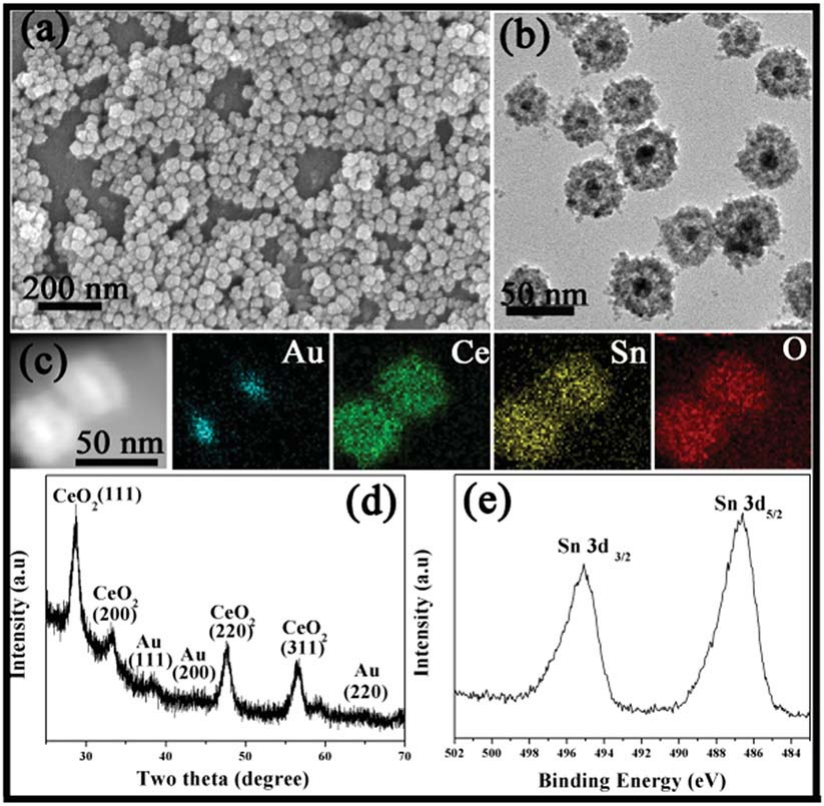
(a) SEM image, (b) TEM image, (c) STEM-EDX elemental maps and (d) XRD pattern of the Au@Ce–Sn MOYSNs. (e) High-resolution XPS for Sn 3d.

Similarly, such a simple strategy can also be applied to the Au@Ce–Sn system. The SEM image in Fig. 2a reveals the uniform sphere structure of the sample. However, the average diameter of the nanospheres is around 35 nm, which is obviously smaller than that of Au@Co–Fe or Au@Co–Ce samples. Furthermore, the TEM image in Fig. 2b displays the obvious presence of a void between the core and shell. The average diameter of 15 nm of the Au core in the Au@Ce–Sn system is bigger than that in the core@shell sample prepared in our previous report.^14^ This is because of the fact that the higher synthesis temperature of the Au@Ce–Sn system can result in a bigger Au core in the original redox assembly stage.^23^ Furthermore, the EDX elemental mapping in Fig. 2c confirms the uniform distribution of Ce and Sn in the whole shell. And the ICP results show that the average contents of Ce and Sn are 57.0 and 20.0 wt%, respectively. Furthermore, all peaks in the XRD pattern (Fig. 2d) can be perfectly indexed to metallic Au and CeO_2_. The high-resolution XPS spectrum (Fig. 2e) of Sn shows two peaks at 486.1 and 494.6 eV, which are the characteristic peaks of Sn 3d_5/2_ and Sn 3d_3/2_ for SnO_2_, respectively.^21^,^22^

**Fig. 3:**
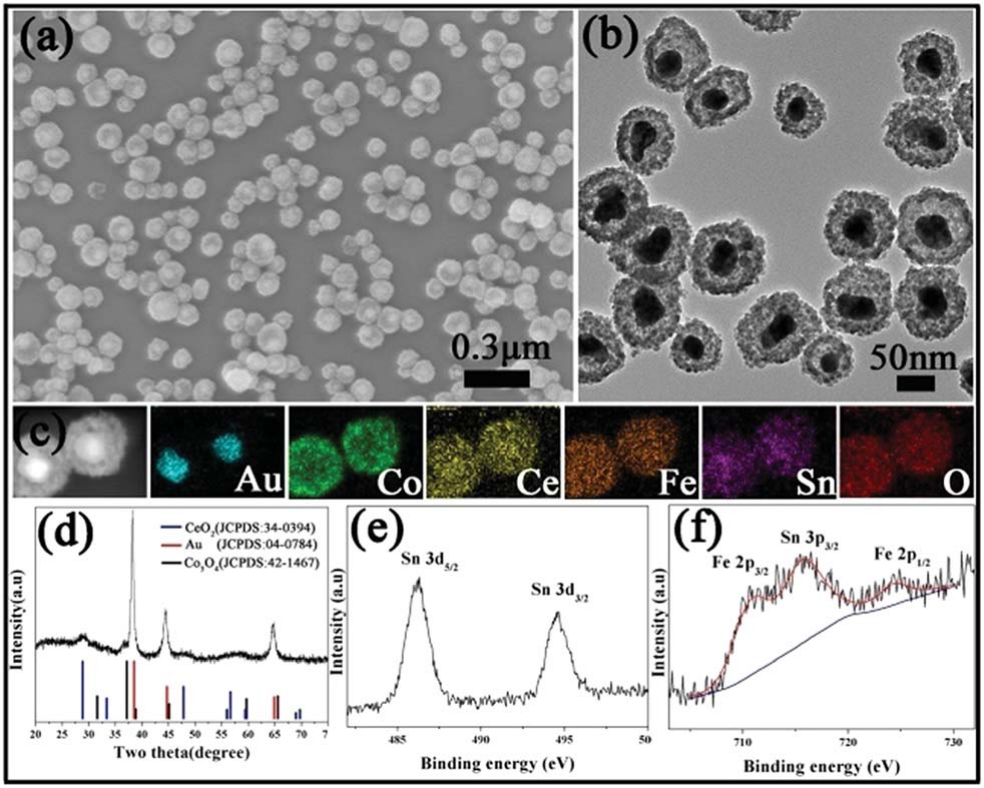
(a) SEM image, (b) TEM image, (c) STEM-EDX elemental maps, and (d) XRD pattern of the Au@Co–Ce–Fe–Sn MOYSNs. High-resolution XPS for (e) Sn 3d and (f) Fe 2p and Sn 3p.

We also attempted to fabricate YSNs containing more than two kinds of oxides in the shell by this simple method. By adding Ce(NO_3_)_3_/FeCl_2_ or Ce(NO_3_)_3_/FeCl_2_/SnCl_2_ solution into the original mixture solution of Au@Co_3_O_4_, Au@Co–Ce–Fe or Au@Co–Ce–Fe–Sn YSNs with multiple oxides in the shell can be obtained. The detailed information of Au@Co–Ce–Fe YSNs is shown in Fig. S5.† The SEM and TEM images (Fig. 3a and b) show that the structure and morphology of Au@Co–Ce–Fe–Sn are similar to those of Au@Co–Fe or Au@Co–Ce samples. The EDX elemental mapping (Fig. 3c) confirms the presence and distribution of Au, Co, Ce, Fe and Sn. The ICP results show that the average contents of Co, Ce, Fe and Sn in samples are 23.9, 15.66, 8.85 and 7.7 wt%, respectively. Combined with the ICP results, the diffraction peaks of the XRD pattern (Fig. 3d) are mainly indexed to Au, Co_3_O_4_ and CeO_2_. There are no apparent peaks of SnO_2_ and Fe_2_O_3_ in the XRD pattern. This might be attributed to the fact that the Fe and Sn oxides formed are highly dispersed in the interstices of the CeO_2_ and Co_3_O_4_ nanoparticles due to the stronger reducing ability of Fe^2+^ and Sn^2+^ ions. The high dispersity of various metal oxides can cause the mutual inhibition of crystal growth during the annealing process.^24^ Therefore, the sample was further analyzed by XPS. The high-resolution XPS spectrum (Fig. 3e) of Sn shows three peaks at 486, 494.5 and 715.7 eV which are the characteristic peaks of Sn 3d_5/2_, Sn 3d_3/2_ and Sn 3p_3/2_ for SnO_2_, respectively.^21^,^22^ In Fig. 3f, the characteristic peaks of Fe 2p_3/2_ and Fe 2p_1/2_ for Fe_2_O_3_ are observed at the binding energies of 710.7 and 724.3 eV, respectively.^21^,^22^

According to previous reports, metal oxides have strong synergistic effects with noble metals to lead to excellent catalytic activity for CO oxidation.^25^–^29^ Furthermore, the strong interaction between CeO_2_ and Co_3_O_4_ can also result in improvement of the catalytic performance.^29^ It is expected that the catalytic activity of MOYSNs can be optimized by altering the relative proportion of different oxide compositions. Additionally, previous reports also show that the Cl^–^ ion has a disadvantageous effect on catalytic CO oxidation.^30^ Therefore, the Au@Co–Ce system was chosen as the catalyst due to no addition of HCl solution and the controllable relative ratio of Co and Ce oxide in its synthesis process. The relative proportion of Co and Ce oxides was altered by controlling the extent of etching. Five samples (without annealing) were obtained by this method. Sample 1 is the Au@Co_3_O_4_ core@shell nanostructure without etching (Fig. S2†). The samples 2–5 are Au@Co–Ce nanospheres with different extents of etching. The contents of Co from sample 2 to sample 4 are gradually reduced (TEM image of Fig. S6† for sample 2, Fig. 1e for sample 3 and Fig. S7† for sample 4). Sample 5 was obtained through severely etching Au@Co_3_O_4_ (Fig. S8†). A massive removal of Co oxide was accomplished, and only about 2% Co remained as shown by ICP analysis. And the detailed content data of the Au, Co and Ce elements for samples 1–7 are listed in Table S1.† In their structure, samples 1 and 2 are core–shell structures and samples 3–5 are yolk@shell structures. Sample 6 is the Au–Co–Ce mixture with the same mass percent of Au, Co and Ce as in sample 4, obtained by directly mixing similar amounts of Au, bare CeO_2_ and Co_3_O_4_ nanoparticles together (Fig. S9†). Sample 7 is a Co–Ce mixture prepared by a co-precipitation process (Fig. S10†).

**Fig. 4:**
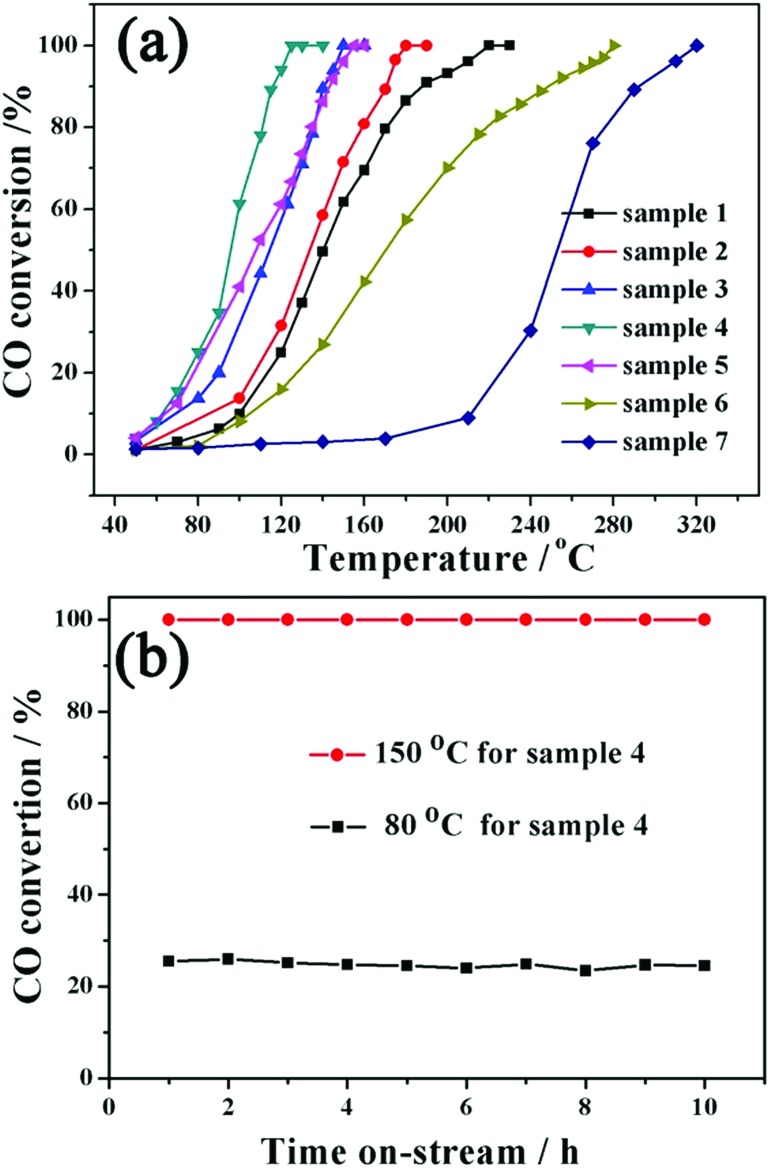
(a) Catalytic activity of samples 1–7 for CO oxidation. (b) Stability test of sample 4 at 150 °C and 80 °C.

Fig. 4a shows the typical CO conversion profiles of the seven samples as a function of temperature. It can be observed that the complete CO conversion temperature for samples 1–7 is approximately 220, 180, 150, 125, 155, 280 and 320 °C, respectively. The Au content values for samples 1–6 are very close to each other; therefore, the differences in the catalytic performance caused by the Au content can be ignored. Two obvious changes can be observed from sample 1 to sample 5, which might be the direct reasons for different catalytic activities of these samples. The first is the change of structure, varying from core@shell structure (samples 1 and 2) to yolk@shell structure (samples 3–5). It is expected that the large void and penetrable shell in the yolk@shell structure enable the better contact of active sites with gas molecules, further resulting in the higher mass-transfer rates and enhanced catalytic activity.^18^,^31^ Therefore, the structural advantage for heterogeneous catalysis might be present in yolk@shell samples. The second change is the oxide component, which might be another important reason for the observed catalytic results. In comparison with single Co_3_O_4_ or CeO_2_ as the support for noble metals, the Co–Ce binary oxide support possesses an additional synergistic effect between Ce and Co oxides.^32^–^34^ Specifically, the synergistic effect between CeO_2_ and Co_3_O_4_ might greatly promote the active oxygen migration, further leading to improved catalytic activity.^32^–^34^ In our Au@Co–Ce system, accompanied by the progress of the etching reaction, the mutual dispersity between Ce and Co oxides is gradually increased from sample 2 to sample 4 (sample 1 without etching). Actually, with the progress of the etching reaction, the distribution of Ce is altered from the outermost shell (Fig. S1b† inset) to the whole shell (Fig. S1d† inset). It can be understood that compared to simple deposition on the surface, the *in situ* redox etching reaction can greatly promote the mutual dispersity of Co and Ce oxide. According to previous literature, the higher mutual dispersity between Ce and Co oxides can produce a stronger synergistic effect and higher catalytic activity.^32^–^34^ Therefore, the gradual enhancement of catalytic activity from sample 1 to sample 4 might be attributed to the advantage of the yolk@shell structure and gradually enhanced synergistic effect of Ce and Co oxide. The interaction of Ce and Co is relatively weak in sample 5 due to the massive removal of Co oxide, further resulting in its decreased catalytic activity. The lower catalytic activity of sample 6 than samples 1–5 can be ascribed to the weak interaction and poor dispersion of the three components. For sample 7, the lowest catalytic activity was observed. The H_2_-TPR of six samples was measured to investigate the interaction between noble metals and metal oxides. The redox ability of metal oxides might be reflected by the H_2_ temperature-programmed reduction reaction (H_2_-TPR). Furthermore, the synergistic effect between noble metals and metal oxides can greatly enhance the redox ability of metal oxides.^33^–^35^ Specifically, the lower reduction peak temperature in the H_2_-TPR curve indicates the stronger redox ability of the sample.^33^,^34^,^36^ It can be found in Fig. S11† that the lowest reduction peak temperature (*T*_lred_) of the Co–Ce mixture (sample 7) is 225 °C. And the Au@Co_3_O_4_ core@shell nanostructure (sample 6) shows the *T*_lred_ at 165 °C. For the Au@Co–Ce nanostructure (samples 2–5), all of the *T*_lred_ values are below 150 °C. Therefore, the synergistic effect between noble metals and metal oxides exists in both core@shell and yolk@shell samples. Furthermore, stability is another important indicator for the evaluation of the catalyst performance. As shown in Fig. 4b, no deactivation occurs for sample 4 when the catalytic reaction is performed at 150 and 80 °C for 10 h. The TEM image (Fig. S12†) further shows that there are no obvious changes in the structure of sample 4 after a long-term catalytic reaction. All results clearly show that by altering the relative content of different oxides, the catalytic performance of the nanospheres can be optimized, and the catalyst is stable and active under long-term catalytic conditions.

## Conclusions

We have developed a general one-pot strategy for the synthesis of well-defined Au@multi-M_*x*_O_*y*_ (Co, Ce, Fe, and Sn) MOYSNs. This method involves the integration of the redox self-assembly process and redox etching process. Furthermore, in tests of CO oxidation, the Au@Co–Ce system was exploited to investigate the effects of oxide composition on its catalytic performance. It was found that the catalytic activity of Au@Co–Ce MOYSNs can be optimized by altering the relative proportion of Co and Ce oxide. Our strategy may provide a new avenue for a facile and clean synthesis of complex noble metal@multi-M_*x*_O_*y*_ MOYSNs with tunable functional materials.

## Experimental section

### Au@Co_3_O_4_ core@shell nanospheres (sample 1)

420 μL of HAuCl_4_ (0.024 M) and 1.6 mL of Co(NO_3_)_2_ (0.1 M) were added into 50 mL of H_2_O and the solution was heated to 70 °C with continuous stirring. Then 3 mL of freshly prepared ammonia solution (20 μL 25%–28% ammonia dissolved in 3 mL of H_2_O) was rapidly added into the mixture solution and the whole system was kept stirring for 10 min. The transparent solution turned black immediately after the addition of ammonia. Finally, the products were separated from the mixture by centrifugation, and washed several times with water and ethanol. After drying at 60 °C, the sample was used to evaluate the catalytic performance without annealing.

### Preparation of Au@Co–Ce MOYSNs (samples 2–4)

420 μL of HAuCl_4_ (0.024 M) and 1.6 mL of Co(NO_3_)_2_ (0.1 M) were added into 50 mL of H_2_O and the solution was heated to 70 °C with continuous stirring. Then 3 mL of freshly prepared ammonia solution (26 μL 25–28% ammonia dissolved in 3 mL of H_2_O) was rapidly added into the mixture solution. After stirring for 2 min, 4 mL of Ce(NO_3_)_3_ (0.02 M) was rapidly added and the whole system was kept stirring for 5 s for sample 2, 10 min for sample 3 and 20 min for sample 4. Then, the products were separated from the mixture by centrifugation, and washed several times with water and ethanol. The products were annealed at 200 °C for 8 h and then at 500 °C for 2 h with a heating rate of 1 °C min^–1^ for further crystallization. The samples were used to evaluate the catalytic performance without annealing.

### Preparation of Au@Co–Fe MOYSNs

420 μL of HAuCl_4_ (0.024 M) and 1.6 mL of Co(NO_3_)_2_ (0.1 M) were added into 50 mL of H_2_O and the mixture was heated to 60 °C. Then 3 mL of freshly prepared ammonia aqueous solution (28 μL 25–28% ammonia dissolved in 3 mL of H_2_O) was rapidly added into the mixture solution. After stirring for 4 min, 3 mL of FeCl_2_ (0.02 M) was rapidly added and the whole system was kept stirring for 4 min. The washing and annealing processes of the sample were similar as the Au@Co–Ce. FeCl_2_solution (0.02 M) was prepared by dissolving 0.1 g of FeCl_2_·4H_2_O into 25 mL HCl solution (containing 100 μL 36–38% HCl solution).

### Preparation of Au@Ce–Sn MOYSNs

600 μL of HAuCl_4_ (0.024 M) and 2.8 mL of Ce(NO_3_)_3_·6H_2_O (0.1 M) were added into 50 mL of H_2_O at 70 °C with continuous stirring. Then 3 mL of freshly prepared ammonia solution (60 μL of 25–28% ammonia dissolved in 3 mL of H_2_O) was rapidly added into the mixture solution and simultaneous timing was started. After stirring for 20 s, 3 mL of SnCl_2_ (0.1 M) aqueous solution was rapidly added and kept for 15 min. Finally, nanoparticles were separated by centrifugation (11 000 rpm and 20 min) and washed with ethanol. SnCl_2_ solution (0.02 M) was prepared by dissolving 0.1125 g of SnCl_2_·2H_2_O into 25 mL HCl solution (containing 200 μL 36–38% HCl solution).

### Preparation of Au@Co–Ce–Fe and Au@Co–Ce–Fe–Sn MOYSNs

420 μL of HAuCl_4_ (0.024 M) and 1.6 mL of Co(NO_3_)_2_ (0.1 M) were added into 50 mL of H_2_O and the mixture was heated to 70 °C. Then 3 mL of freshly prepared ammonia aqueous solution (24 μL 25–28% ammonia dissolved in 3 mL of H_2_O) was rapidly added into the mixture solution. After stirring for 4 min, firstly, 1.5 mL of Ce(NO_3_)_3_ (0.02 M) was rapidly added. After stirring for 3 min, 1.5 mL of FeCl_2_ (0.02 M; for Au@Co–Ce–Fe) or the mixture solution (containing 1 mL of FeCl_2_ of 0.02 M and 0.5 mL of SnCl_2_ of 0.02 M; for Au@Co–Ce–Fe–Sn) was rapidly added, and then the whole system was kept stirring for 13 min. The washing and annealing processes of the samples are the same as those of Au@Co–Ce.

### Preparation of sample 5

420 μL of HAuCl_4_ (0.024 M) and 1.6 mL of Co(NO_3_)_2_ (0.1 M) were added into 50 mL of H_2_O and the solution was heated to 75 °C with continuous stirring. Then 3 mL of freshly prepared ammonia solution (20 μL 25–28% ammonia dissolved in 3 mL of H_2_O) was rapidly added into the mixture solution. After stirring for 10 s, 6 mL of Ce(NO_3_)_3_ (0.02 M) was rapidly added and the whole system was kept stirring for 30 min. The nanoparticles were separated from the mixture by centrifugation, and washed several times with water and ethanol. After drying at 60 °C, the sample was used to evaluate the catalytic performance without annealing.

### Preparation of Au–Co–Ce mixture sample 6

#### Au nanoparticles

50 mg Au@Co–Fe yolk@shell nanospheres (without annealing) were added into 50 mL acetic acid (99.8%) and stirred at 60 °C for 10 h. Finally, the Au nanoparticles were obtained by washing several times with ethanol.

#### CeO_2_ nanoparticles

10 mL of Ce(NO_3_)_3_ (0.02 M) was added to 10 mL of H_2_O, and then 2 mL of NaOH (0.2 M) aqueous solution was rapidly added. The solution was stirred at 70 °C for 30 min. The sample was purified by centrifugation and washed with water.

#### Co oxide

4 mL of Co(NO_3_)_2_ (0.1 M) was added into 50 mL of H_2_O and the solution was heated to 70 °C with stirring. Then 4 mL of mixture solution (containing 100 μL of 25–28% ammonia, 50 μL of 30% H_2_O_2_ and 3.85 mL of H_2_O) was added and the whole solution was kept stirring for 30 min. The sample was purified by centrifugation and washed with water.

#### Au–Co–Ce mixture

The nanoparticles of Au, CeO_2_ and Co oxide were mixed together with the same mass ratio as in sample 3. After ultrasonic treatment for 10 min in ethanol, the sample was dried at 60 °C.

### Preparation of sample 7

10 mL of Ce(NO_3_)_3_ (0.1 M) and 5.8 mL of Co(NO_3_)_2_ (0.1 M) were added to 50 mL of H_2_O at 60 °C, and then 4 mL of NaOH (2 M) aqueous solution was rapidly added and the whole solution was kept stirring for 30 min. The sample was washed with water and dried.

### Characterization

X-ray diffraction (XRD) was performed on a Rigaku-D/max 2500 V X-ray diffractometer with Cu-Kα radiation (*λ* = 1.5418 Å). The morphologies of the products were directly examined by scanning electron microscopy (SEM) using a HITACHI S-4800 instrument at an accelerating voltage of 20 kV. Transmission electron microscopy (TEM) images were obtained with a TECNAI G2 high-resolution transmission electron microscope, operating at 200 kV. XPS measurements were performed on an ESCALAB-MKII250 photoelectron spectrometer (VG Co.) with Al Kα X-ray radiation as the X-ray source for excitation. Inductively coupled plasma (ICP) analyses were performed with a Varian Liberty 200 spectrophotometer to determine the contents. H_2_-TPR measurements were performed in a conventional flow apparatus. 10% H_2_/He flow was passed over the catalyst bed while the temperature was ramped from 100 °C to 800 °C at a heating rate of 5 °C min^–1^. The hydrogen consumption signal was monitored by a thermal conductivity detector (TCD).

### CO catalytic oxidation

30 mg of catalyst was put into a stainless steel reaction tube. The experiment was carried out under a flow of the reactant gas mixture (1% CO, 20% O_2_, balance N_2_) at a rate of 30 mL min^–1^. The composition of the gas was monitored online by gas chromatography (GC 9800).

## Conflicts of interest

There are no conflicts to declare.

## Supplementary Material

Supplementary information
