# Supplementary material for: A general one-pot strategy for the synthesis of Au@multi-oxide yolk@shell nanospheres with enhanced catalytic performance
Source: Chem Sci. 2018 Aug 6;9(38):7569–74. doi: 10.1039/c8sc01520a (PMC6180307; doi:10.1039/c8sc01520a)
Supplement: Supplementary file 1 [file SC-009-C8SC01520A-s001.pdf]

Electronic Supplementary Material (ESI) for Chemical Science.
This journal is © The Royal Society of Chemistry 2018

Electronic Supplementary Information

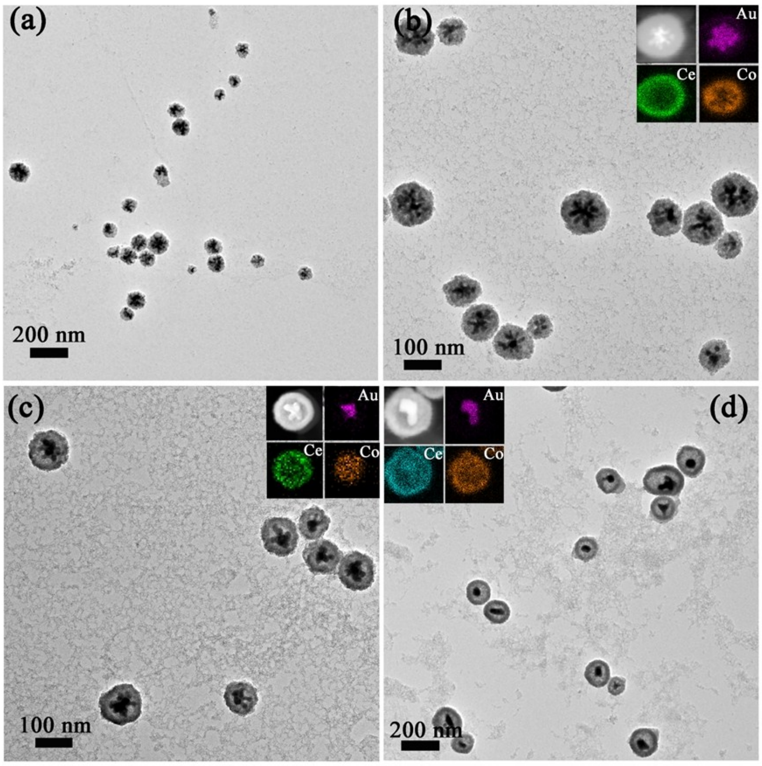

Fig. S1. (a-d) TEM images of Au@Co-Ce prepared at different reaction times: a) 120 s, b) 125 s, c) 4 min, d) 10 min; The insets show STEM-EDX elemental maps of corresponding samples.

The addition of ammonia can trigger the redox self-assembly process to result in formation of the Au@Co_3O_4 multicore-shell nanospheres (Fig. S1a) after reacting for 120s. When $Ce(NO_3)_3$ is added at 120 s, Ce^{3+} rapidly reacts with the Co_3O_4 on the surface to form CeO_2 on the outermost shell. After etching with Ce^{3+} for 5 s, the core-shell structure remain unchanged (Fig. S1b). However, the EDX element mapping (Fig. S1b inset) reveals the CeO_2 is already form on the outermost shell. As the reaction proceeds, the electrons released from Ce^{3+} migrate onto the Au surface to etch the Co_3O_4 around Au NPs.[1] Accompanied by the dissolution of Co_3O_4 and driven by thermodynamic equilibrium, the Au NPs begin to aggregate gradually, and the small void spaces around Au form slowly (Fig. S1c). In addition, because the shell is a stack of metal oxide particles, many of the pinholes should be present in the

interstice of the metal oxide particles. These pinholes not only help Ce^{3+} to diffuse into the interior of the nanosphere but also improve the dissolution of the internal Co_3O_4.[1] After etching for 10 min, the apparent yolk-shell structure and large Au core (Fig. S1d) are formed due to the massive dissolution of Co_3O_4.

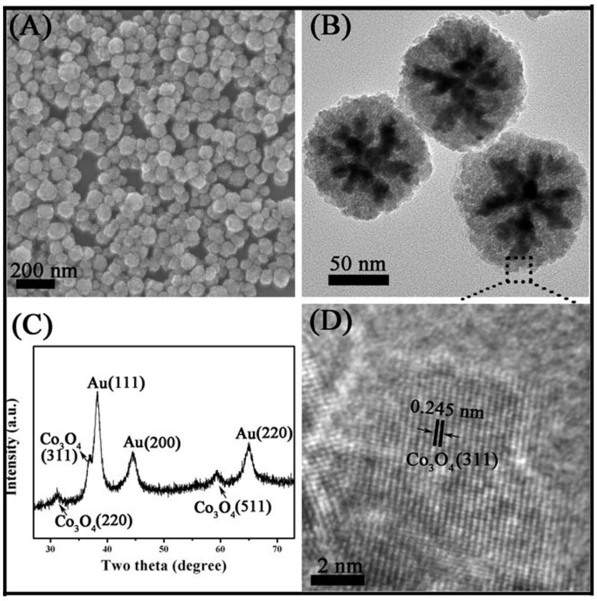

Fig. S2. A) SEM image, B) TEM image, C) XRD pattern and D) HRTEM image of Au@Co_3O_4 multicore-shell nanosphere.

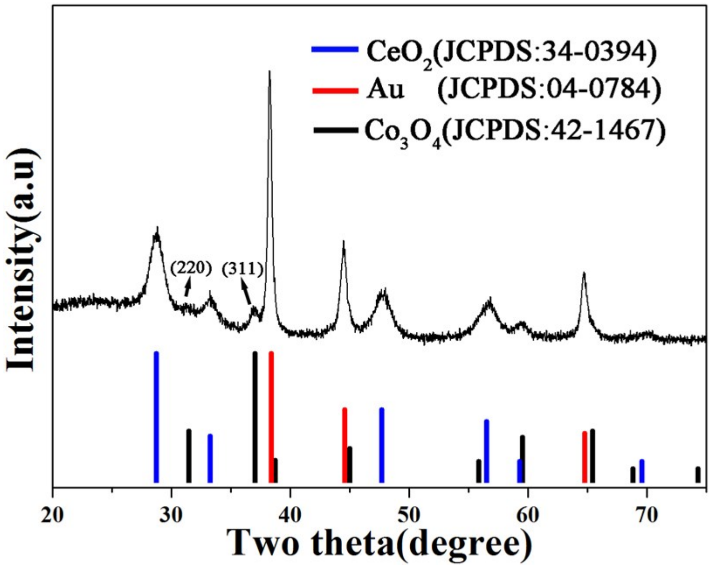

Fig. S3. XRD pattern of Au@Co-Ce MOYSNs.

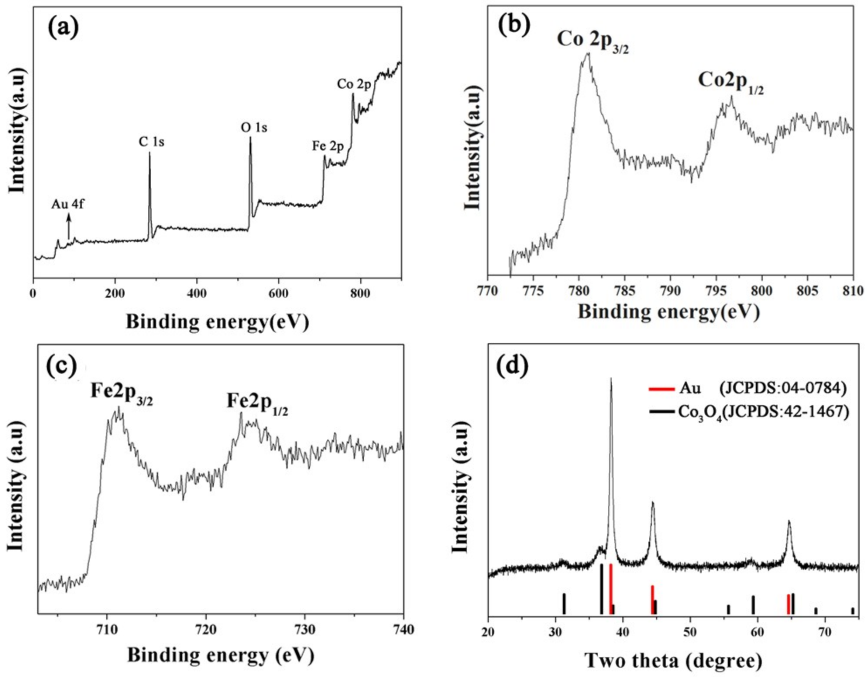

Fig. S4. (a) XPS, (b), (c) high-resolution XPS for (b) Co 2p and (c) Fe 2p, (d) XRD pattern of Au@Co-Fe MOYSNs.

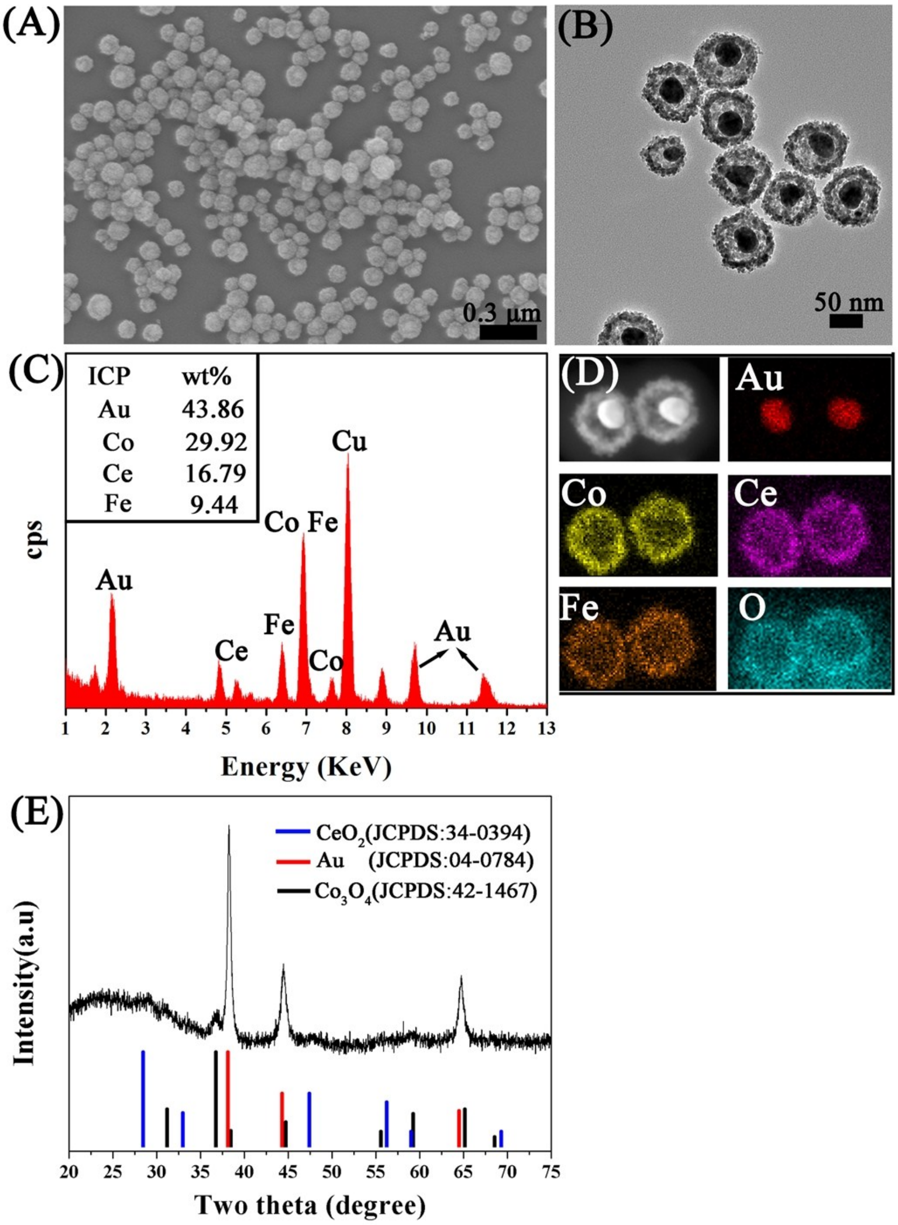

Fig. S5. A) SEM image, B) TEM image, C) STEM-EDX profile, the inset is the result of ICP, D) STEM-EDX elemental maps, E) XRD pattern of Au@Co_3O_4/CeO_2/Fe_2O_3 sample.

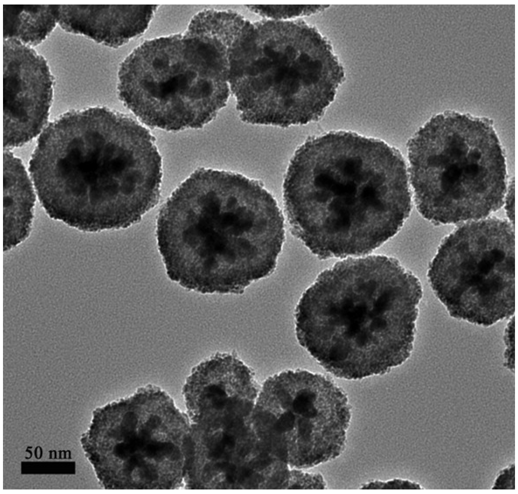

Fig. S6. TEM image of sample 2 (Au@Co-Ce multicore@shell nanosphere)

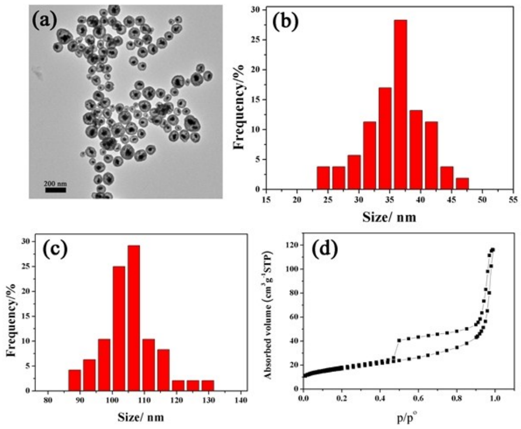

Fig. S7. a) TEM image, b) Au size distribution histogram, c) nanosphere size distribution histogram, d) N_2 adsorption-desorption isotherms of sample 4

The results show that the BET surface area of sample 4 is 59.8 m2 g−1 and pore diameter is 19.53 nm.

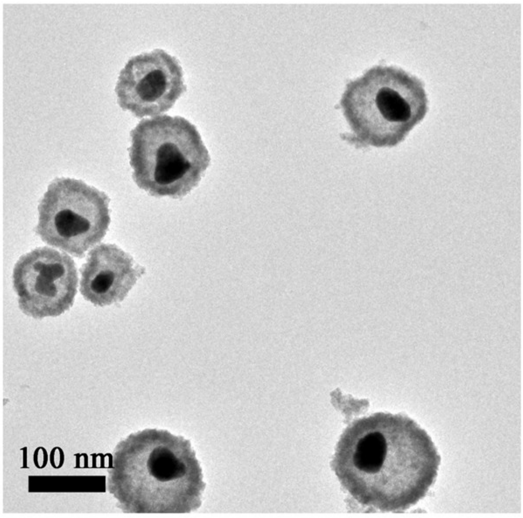

Fig. S8. TEM image of sample 5.

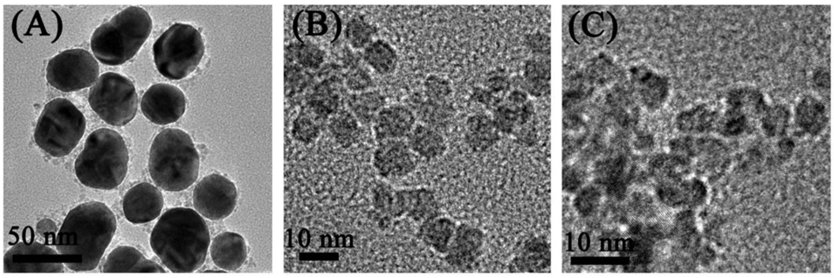

Fig. S9. TEM images of (A) Au nanoparticle, (B) Co_3O_4, (C) CeO_2 nanoparticles.

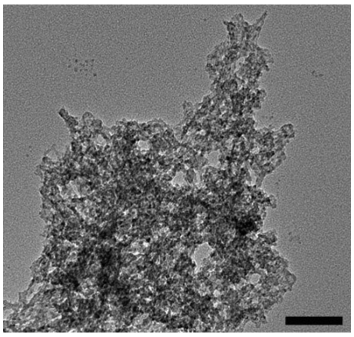

Fig. S10. TEM images of Co-Ce mixture prepared by co-precipitation process.

ICP /wt%	Sample 1	Sample 2	Sample 3	Sample 4	Sample 5	Sample 7
Au	33.2%	32.3%	32.8%	33.3%	31.6%	0%
Co	65.8%	32.6%	19.8%	13.2%	2.4%	19.4%
Ce	0.0%	35.1%	47.4%	53.5%	66.0%	80.6%

Table S1. ICP results of Sample 1-5 and 7.

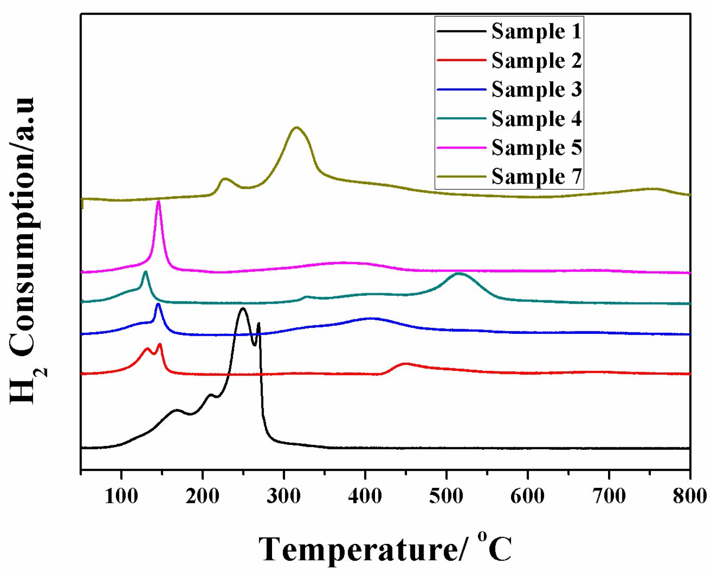

Figure S11. The H_2-TPR curves of sample 1-5 and sample 7.

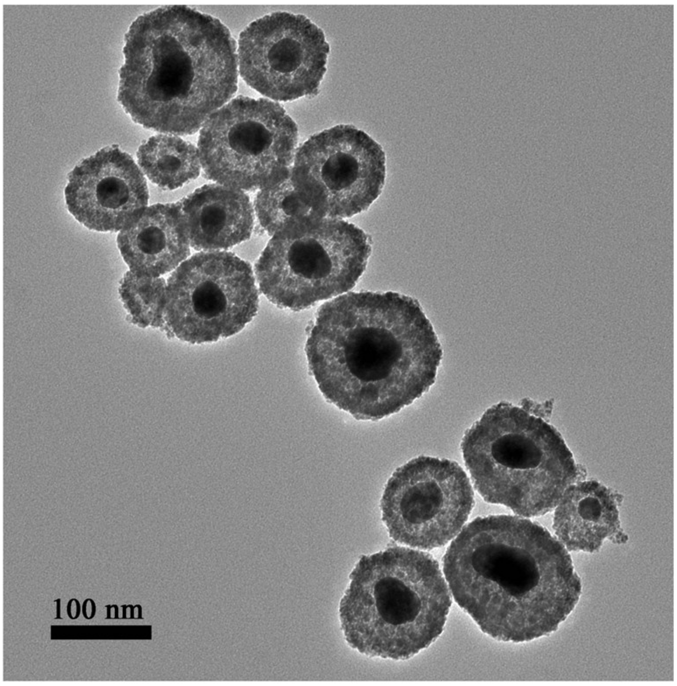

Fig. S12. TEM image of sample 4 after catalytic test

Catalyst	Amount (mg)	Size of Au (nm)	Temperature for 100 % CO conversion(^{o}C)	space velocity ($cm^3 \cdot h^{-1} \cdot g^{-1}$)	reference
Au@CeO_2core-shell	200	17	155	15000	ref. 2
Au@ZrO_2 yolk-shell	50	15-17	240	80000	ref. 3
Au@SnO_2 yolk-shell	50	40	230 (for 50%)	84000	ref. 4
Au@CeO_2/ZrO_2yolk-shell	none	3-5	120	120000	ref. 5
Au@SiO_2 core-shell	30	1.5	180	30000	ref. 6
Au@MnO_2 yolk-shell	30	10-15	130	60000	ref. 7
Au@Co-Ce yolk-shell	30	35	125	60000	this work

Table S2. Comparison of the Size of Au, temperature for 100 % CO conversion and space velocity for CO oxidation with other oxide-encapsulated Au catalysts.

Reference:

1. M. H. Oh, T. Yu, S.-H. Yu, B. Lim, K.-T. Ko, M.-G. Willinger, D.-H. Seo, B. H. Kim, M. G. Cho, J.-H. Park, K. Kang, Y.-E. Sung, N. Pinna and T. Hyeon, *Science*, 2013, **340**, 964.
2. J. Qi, J. Chen, G. Li, S. Li, Y. Gao and Z. Tang, *Energy & Environmental Science*, 2012, **5**, 8937.
3. P. M. Arnal, M. Comotti and F. Schüth, *Angewandte Chemie*, 2006, **118**, 8404.
4. K. Yu, Z. Wu, Q. Zhao, B. Li, and Y. Xie, *J. Phys. Chem. C*, 2008, **112**, 2245.
5. C. Du, Y. Guo, Y. Guo, X.-Q. Gong and G. Lu, *Journal of Materials Chemistry A*, 2017, **5**, 5601.
6. T. Zhang, H. Zhao, S. He, K. Liu, H. Liu, Y. Yin, and C. Gao, ACS nano, 2014, 8, 7297.
7. M. Gong, J. Zhang and C.-A. Wang, *Chemistry Letters*, 2017, **46**, 876.
